# Quality of Childbirth Care in Women Undergoing Labour: Satisfaction with Care Received and How It Changes over Time

**DOI:** 10.3390/jcm8040434

**Published:** 2019-03-29

**Authors:** Miriam Donate-Manzanares, Teresa Rodríguez-Cano, Juan Gómez-Salgado, Julián Rodríguez-Almagro, Antonio Hernández-Martínez, Ester Barrilero-Fernández, Luis Beato-Fernández

**Affiliations:** 1 Virgen de la Salud Hospital, 45004 Toledo, Spain; m_donate_manzanares@hotmail.com; 2 Department of Psychiatry, Hospital General Universitario, 13005 Ciudad Real, Spain; trcano@gmail.com (T.R.-C.); lbeatof@gmail.com (L.B.-F.); 3 Department of Sociology, Social Work and Public Health, University of Huelva, 21071 Huelva, Spain; salgado@uhu.es; 4 Safety and Health Posgrade Program, Universidad Espíritu Santo, Guayaquil 091650, Ecuador; 5 Department of Nursing of University of Castilla la Mancha, 13071 Ciudad Real, Spain; 6 Mancha-Centro Hospital, Alcázar de San Juan, 13600 Ciudad Real, Spain; antomatron@gmail.com; 7 Hospital General Universitario, 13005 Ciudad Real, Spain; ester.barrilero@hotmail.com

**Keywords:** childbirth, patient satisfaction, surveys and questionnaires, observational study, quality improvement

## Abstract

(1) Background: To evaluate women’s satisfaction with the care received during childbirth undergoing labour and how this changes over time. (2) Methods: An observational, multicentre and longitudinal study in two public hospitals of Spain with a convenience sample of 248 women during the post-partum period. Satisfaction was evaluated using the Intrapartal-Specific Quality from the Patient’s Perspective questionnaire. Socio-demographic and clinical variables were considered. A bivariate analysis evaluated the relationship between satisfaction and the recorded variables to compare the hypotheses, and also to know if there were any significant differences between the scores obtained at the various evaluated time points (three days, 15 days, one year postpartum). The multivariate analysis by binary logistic regression helped complete the study about the variables related to the level of satisfaction; (3) Results: The mean scores for satisfaction at each time point were high, and they lowered with time. The bivariate analysis showed an association between satisfaction and the following variables: ‘mother’s place of birth’, ‘level of education’, ‘type of labour onset’, ‘type of birth’ and ‘type of perineal trauma’. For the multivariate analysis, women who gave birth vaginally, began labour spontaneously and had an epidural were more satisfied than those who did not; (4) Conclusions: Women seemed to obtain good levels of satisfaction with the care they received. Even so, based on continuously improved quality, we proposed some improvement measures, particularly regarding the physical environment and the information offered to patients. It is important to know at what time experience while giving birth should be evaluated as scores can lower with time as part of its normal evolution.

## 1. Introduction

Quality care is a complex and hard phenomenon to define [1]. One of the most widespread descriptions was made by the WHO: “the extent to which health care services provided to individuals and patient populations improve desired health outcomes. In order to achieve this, health care needs to be safe, effective, timely, efficient, equitable, and people-centred” [2]. One relevant part of this perspective refers to how care can be patient-centred, that is, users’ preferences must be borne in mind, as well as their experience and their satisfaction with care. Hence, the WHO states that assessing women’s satisfaction with the care they receive during childbirth is a way to improve the quality of the rendered health services [3].

Many studies have associated childbirth experiences with their effect on women’s health and their environment. Indeed, problems like depression in future pregnancies [4], postpartum depression [5], anxiety [6] and post-traumatic stress syndrome [7,8] have been exacerbated due to negative childbirth experiences.

In Spain, childbirth care has become much more technical in recent decades. However, this does not necessarily mean that the quality of the provided services has improved, as many new techniques have been introduced without much significant scientific evidence that backs them up [9]. Such technification also leads to the belief that the childbirth process is something that must be strictly controlled by healthcare professionals, establishing a distance with the mothers’ active role [10]. In response to this, women’s associations have come together to demand a change in the care currently received during childbirth (e.g., Asociación El Parto es Nuestro [11]), and some organisations in both Spain and elsewhere have drawn up Practical Guides on this matter (e.g., WHO, 1996 [12]; Spanish Ministry of Health and Social Policy, 2010 [9]) in an attempt to regulate medicalisation and interventions during childbirth.

The aim of this study was to know if women’s satisfaction with the care received during childbirth undergoing labour was similar to the results obtained in other studies in different settings. Our study attempts to overcome these limitations and to widen knowledge on the childbirth experience by analysing satisfaction with childbirth.

## 2. Materials and Methods

### 2.1. Design and Sample

The scope of this observational, multicentre and longitudinal study corresponded to the Hospital General Universitario de Ciudad Real and the Hospital Virgen de la Salud, both tertiary public hospitals of the region of Castilla-La Mancha. These hospitals have a high health care burden. One of these hospitals is located at Level 3 (1200–2400 births/year) and the other at Level 4 (more than 2400 births/year) in the Classification by Number of Births (Strategy for Care with Normal Births, 2007. The Spanish Ministry of Health and Consumption). Both hospitals have health sciences students carrying out their practical training in them.

The inclusion criteria for this study were: being older than 18 years; being able to understand Spanish; being able to read and write. Any women who underwent a scheduled caesarean or an emergency caesarean before labour was excluded.

To estimate the sample size, Freeman’s criteria [13] were followed, according to which 10 participants are needed for each independent variable included in the binary logistic regression model. In our case, eight variables were included in this model. Thus, the required sample size had to include a minimum of 80 women. This way, the number of participants included was enough for the study.

When evaluating patients’ satisfaction, the established aim was to obtain the largest possible number of participants during the time period available.

### 2.2. Measures

#### Intrapartal-Specific Quality from the Patient’s Perspective Questionnaire (QPP-I)

The Intrapartal-Specific Quality from the Patient’s Perspective Questionnaire (QPP-I) [14] measures women’s satisfaction with their childbirth experience when the aim is a vaginal delivery by including a wide range of aspects that can affect the process. This scale has been validated by its authors in Sweden, and is also supported by a comprehensive preliminary study that includes qualitative techniques that make it more complete [15]. It has been translated into Spanish and transculturally adapted to the context, and its psychometric properties obtained good scores [16]. During the transcultural adaptation of the QPP-I to the Spanish context, using 11 dimensions with 39 items seemed more suitable according to the exploratory factor analysis. The original scale included 10 dimensions with 40 items. Item 3 (electronic devices) was eliminated as it obtained a low level of saturation, and the explained variance was 70.5% when this item was removed. Validity was evaluated by comparison with another validated tool, the COMFORTS scale, with an area under the ROC curve of 0.82 (95%CI: 0.76–0.88). For reliability, the Cronbach’s α index gave a value of 0.92, and that of the intraclass correlation index in the test-retest was 0.76 (95%CI: 0.65–0.84).

The QPP-I adapted to the Spanish context is made up of 39 items distributed over 11 dimensions: (1) “the work of midwives” (five items); (2) “second stage of labour and evaluation of birth” (six items); (3) “the work of doctors” (four items); (4) “the work of nursing assistants” (three items); (5) “intervention and decision making” (five items); (6) “accompaniment” (four items); (7) “information” (four items); (8) “facilities” (two items); (9) “close relatives” (two items); (10) “layout of the room” (two items); and (11) “feeling of safety” (two items).

To measure the satisfaction with childbirth care, each item was expressed as a statement (e.g., “Midwives treated me with respect”), which participants had to answer according to the statement “this is what I experienced…”. The participants had to give answers by choosing among these options: “do not agree at all” (1); “partly agree” (2); “mostly agree” (3); “fully agree” (4). Therefore, the scores for each item went from 1 to 4. The higher the score for each item, the greater the satisfaction.

All items had a “non-applicable” option.

### 2.3. Socio-Demographic and Clinical Data

The following socio-demographic data were recorded: age, marital status, level of education, mother’s place of birth and whether or not the participant attended antenatal classes.

In the case of attending antenatal classes, the participant was considered to meet this requisite after having attended, at least, six of the eight sessions the training programme for childbirth includes in the public centres of our setting.

The variable “participant’s country of origin” categorised women as Spanish or foreign. This decision was made because the size of the foreign population was particularly small, so we grouped it to get more statistical power.

The recorded clinical data were: parous, gestational age at birth, type of birth, type of labour onset, use of epidural, presence and type of perineal trauma, duration of labour, type of feeding and baby’s weight at birth.

Regarding gestational age, the participants were classified as “to term” (birth after 37 weeks of gestation) and “preterm” (births before 36 + 6 weeks of gestation), since it is the most used classification by other studies and, this way, comparisons with these studies were easier.

### 2.4. Procedures

As for the data collection method, women who met the inclusion criteria were asked if they were willing to participate in the study on post-partum day 3 (while they were still in the postnatal ward). At this time point, women were asked to complete the questionnaire for the first time on that day (day 3). They were sent the questionnaire 15 days after giving birth, and also one year after giving birth. Since no consensus in the literature has been reached about the best time point to evaluate the childbirth experience, these time intervals were selected because they were similar to those used in previous studies [17,18] and were the ones which best matched teamwork requirements.

All the procedures involving human participants met the ethical standards of the institutional and/or national research committee, as well as the 1964 Declaration of Helsinki and its later amendments or comparable ethical standards. Before carrying out the study, the Clinical Research Ethics Committees of both hospitals gave their approval. All the participants received verbal and written information about the study, including the fact that participation was voluntary and they could withdraw from the study at any time with no penalties whatsoever. They received written informed consent forms and answered the questionnaires anonymously. In order to contact them both 15 days and one year after childbirth, they were assigned an alphanumerical code. Their personal data were hidden, except for those data required to contact them, which were known by only one researcher who contacted them 15 days and one year after giving birth.

### 2.5. Data Analysis

To apply the statistical methods, the 24.0. version of SPSS software was used.

The quantitative variables were “participants’ age” and “duration of delivery,” although they were also analysed as qualitative variables, stating the categories included in Table 1 for each of them. The rest of the variables were qualitative.

Absolute frequencies (such as the number total participants in each category) and relative frequencies (like the proportion of each category) were used to describe the qualitative variables. Additionally, the mean and standard deviation (SD) were used to describe the quantitative variables.

Derived from the hypothesis that there are significant differences between satisfaction scores as for the moment when the questionnaire was answered (by first using hypothesis testing for the related samples: Wilcoxon Test), the results obtained at each different moment were compared. A value of *p* < 0.05 was considered to indicate significant differences between groups.

For each time point (three days, 15 days, one year), the relationship between the QPP-I score and the factors believed to be associated from a theoretical viewpoint, such as type of birth or onset of labour, as well as the items corresponding to the feelings experienced by women, as described above, were analysed. Our hypothesis relied on the fact that satisfaction scores varied according to the value each analysed variable had. This was done by a bivariate analysis with hypothesis testing. The Mann–Whitney U test was used to evaluate the relationship between the qualitative dichotomous variables, the Kruskal–Wallis test for the polytomous variables, and the Spearman’s correlation for the quantitative variables.

A multivariate analysis was carried out using binary logistic regression, following the automated backward procedure in the statistics software to control the confounding bias and to determine the net effect of each factor on satisfaction. Odds ratios (OR) were estimated with 95% confidence interval (95% CI). The main variable result was a cut-off point of 3.2 in the QPP-I, given the values for sensitivity (96.8%) and specificity (63.4%) obtained from its application. The independent variables were those that obtained a *p* < 0.05 value in the bivariate analysis (level of education; type of childbirth; type of perineal trauma; type of labour onset; mother’s place of birth) and those which, based on the reviewed literature, were significant due to clinical plausibility (antenatal classes; duration of labour; use of epidural).In the results section, each of the regressions carried out by comparing the initial model (including all the variables analysed) and the final model (just the variables that had a greater effect on the analysis) will be described.

## 3. Results

### 3.1. Descriptive Statistics

From 1 July to 30 September 2016, 329 women were invited to participate in the study, of whom 4% (*n* = 13) chose not to do so. Of those who accepted, 78.5% (*n* = 248) handed in the correctly completed questionnaire. No significant differences were found between these three groups of women (those who declined to participate, those who did not hand in a completed questionnaire and those who handed in a completed questionnaire) in terms of the studied socio-demographic and clinical variables.

Of the 248 who answered the questionnaire three days after childbirth, 36.3% (*n* = 90) correctly handed it in 15 days after giving birth. One year later, this figure was 33.5% (*n* = 83). The great decrease of responses after 15 days may be due to the fact that the participants were at an early adaptation stage and they had less free time to answer after a year. When comparing the women who replied at 15 days and those who did not, a difference was found regarding the age variable (*p* = 0.018). Differences were observed between the women who replied at one year and those who did not for the variables “duration of labour” (*p* = 0.030); “age” (*p* = 0.019); “level of education” (*p* = 0.041). We also made a comparison between each group, that is, the 248 women who replied at three days, the 90 who replied at 15 days, and the 83 who replied at one year. No differences were found for any of the studied variables.

The average age of all the participants (*n* = 248) was 32.3 years (SD: 4.79, range 18–43). Spain was the country of origin for the majority of women (*n* =215, 86.7%). Regarding clinical data, most births were at term (*n* = 239, 96.4%), had a spontaneous onset (*n* = 152, 61.3%) and were eutocic (*n* = 163, 65.7%). The mean score (for which we must bear in mind that the maximum possible value to be scored was 4) of the QPP-I at three days was 3.41 (0.45), 3.40 (0.50) at 15 days, and 3.25 (0.58) at one year.

Table 1 provides details on the socio-demographic and clinical variables, as well as the QPP-I scores, divided into groups.

Table 2 shows the means obtained from the eleven QPP-I dimensions and for all the time points when the questionnaire was administered. The dimensions with the highest satisfaction scores were (1) “the work of midwives,” (2) “second stage of labour and evaluation of birth,” (3) “the work of doctors,” (4) “the work of nursing assistants,” (6) “accompaniment,” (9) “close relatives” and (11) “feeling of safety,” followed by (5) “intervention and decision making,” (7) “information,” (8) “facilities” and (10) “layout of the room.”

### 3.2. Comparative Analysis of the Results Obtained at the Different Time Points

Of the 248 participants who answered the questionnaire at three days, 90 did so again at 15 days. No significant differences were found as for the total results obtained (*p* = 0.609). However, differences were found in dimensions: “(3) the work of doctors” (*p* = 0.033: satisfaction lowered at 15 days), and “(5) intervention/decision making” (*p* = 0.002: satisfaction increased at 15 days).

At one year, 83 women answered the questionnaire. Significant differences were found when comparing the total results with the results obtained at three days (*p* = 0.03: satisfaction had decreased at one year). The dimensions in which the differences were found were (1) (*p* = 0.001), (2) (*p* = 0.026), (3) (*p* = 0.002), (4) (*p* = 0.000), (5) (*p* = 0.016), (6) (*p* = 0.033), (9) (*p* = 0.002) and (11) (*p* = 0.001). Satisfaction had decreased in all these dimensions at one year, except for factor (5), for which satisfaction was higher.

Of those who answered at 15 days, 53.3% (*n* = 48) also did so at one year. Significant differences were found between the total results obtained at both time points (*p* = 0.004: satisfaction decreased at one year). On this occasion, differences were found in the dimensions: (1) (*p* = 0.043), (3) (*p* = 0.040), (4) (*p* = 0.006) and (6) (*p* = 0.014). Satisfaction had decreased at one year in all of them.

This information is set out in more detail in Table 1 and Table 2.

### 3.3. Results of the Bivariate and Multivariate Statistical Analyses

In the bivariate analysis, the highest levels of satisfaction at three days after giving birth were associated with being foreign (i.e., not Spanish) (*p* = 0.016), having a low level of education (*p* = 0.022), not having an intact perineum (*p* = 0.035) and, especially, with giving birth vaginally (*p* = 0.000) and with spontaneous labour onset (*p* = 0.001). At 15 days, only the association with the type of labour remained (*p* = 0.023). At one year, the associations with the level of education (*p* = 0.048), intact perineum (*p* = 0.040) and, once again, type of birth, remained (*p* = 0.002). These data are explained in more detail in Table 1.

In the multivariate analysis, the final model obtained using only the variables ‘type of labour’ and ‘type of labour onset’ accounted for 75.4% of the variance at three days post-partum. Compared to the women who had undergone a caesarean, women who had given birth vaginally had an OR of 3.14 times more likely to be satisfied (95% CI: 1.60–6.17). Compared to the women whose birth had been induced, women who spontaneously went into labour had an OR of 2.17 times more likely to be satisfied (95% CI: 1.20–3.93). Table 3 provides further details on this analysis.

At 15 days post-partum, the final model obtained with the variables ‘type of labour’ and ‘use of epidural’ accounted for 73.3% of the variance. Women who used epidural had an OR of 3.76 times more likely to be satisfied (95% CI: 1–14.15) than those who did not. Those who had given birth vaginally had an OR of 3.27 times more likely to be satisfied than those who had a caesarean (95% CI: 1.04–10.27). These data are explained in more detail in Table 4.

At one year, the final model that included ‘type of labour’ as the only significant variable accounted for 66.3% of the variance. Compared to the women who underwent a caesarean, women who had given birth vaginally had an OR of 5.04 times more likely to be satisfied (95% CI: 1.45–17.56). This information is developed in more detail in Table 5.

## 4. Discussion

### 4.1. Satisfaction over Time

There is controversy over the best time point to evaluate women’s satisfaction with their childbirth experience. In general, users’ evaluations vary depending on the time point after childbirth. Bennett [19] described how women became more critical about the procedures followed during their childbirths with time, while Simkin [20] found that the importance they attached to negative events intensified with time. Waldenström [18] compared what women thought at two months post-partum, and then at one year post-partum. This author found that 60% were still of the same opinion, and he pointed out that very early evaluations of childbirth experiences can be influenced by feelings of relief and euphoria. Even so, authors still do not generally agree, and studies differ on how long after childbirth satisfaction should be evaluated [7,17,21]. As there is still great variability on this respect among the different studies, one of the aims of this study was to shed some light on this matter.

In the present study, we saw that the overall evaluation of the childbirth experience decreased over time, which also occurred regarding most dimensions of the QPP-I. In the first post-partum week, only positive consequences were observed, such as having overcome childbirth and assuming a new maternal role. As time passed, problems could arise which were not noticed before or were not expected, such as parenting problems or health problems like post-partum depression or perineal pain from intrapartum injuries [22]. In other health-related fields, something similar occurs:as negative events in the patient’s well-being takes place after receiving care, satisfaction with this care diminishes [23,24].

The only dimension in which increased satisfaction was seen over time was “(5) intervention/decision making,” including items about the degree of interventionism in birth and the extent to which users were able to make decisions about the care they received. At three days post-partum, participants evaluated this dimension with fairly low levels of satisfaction, which meant that they were not satisfied with their own level of participation in the process. These scores increased over time, but remained negative. This change may have been influenced by the paternalistic paradigm. There is still a very deep-rooted collective belief that health professionals always know what the best decision is, without having to offer all the available options [25]. This is one of the changes we are witnessing in the paradigm. Thus, we must facilitate the adoption of a holistic care model that is integral and that brings women back to their main role [10].

### 4.2. Satisfaction with the Care Received during Childbirth

Satisfaction with childbirth care is complex as it involves many variables. Some factors that have been associated with the level of satisfaction are those related to controlling the situation, fulfilling expectations [26], to the care received [17,27], pain during childbirth [28], certain obstetric factors like the type of birth [26,29,30], type of labour onset [29,31] and duration of labour [28,29], as well as attending antenatal classes [32].

Using a multivariate analysis, the variable that showed the clearest influence on satisfaction, and which remained significant at all the subsequently analysed time points, was “type of labour.” Higher levels of satisfaction were obtained in women who had a vaginal birth, which agrees with other studies [26,30]. The variables “type of labour onset” and “use of epidural” also influenced the initial post-partum stages, finding more satisfaction in women who had begun labour spontaneously [31] and those who had used an epidural. The relationship between the use of epidural and maternal satisfaction is controversial among the literature. Some authors have described a worse experience if an epidural is used [31,33], while others have found no relationship [26] and some, including this study, have observed that users who had been administered an epidural reported a better experience [34]. Epidural use has become widespread in Spain since the year 2000, and it has become a usual and beneficial technique in childbirth [35], which could explain our study results.

In the overall evaluation that the participants made of the care received while giving birth, satisfaction was quite high in this sample. Notwithstanding, several dimensions could be improved.

A feeling of safety, the work of professionals, support from family members and the evaluation of labour and childbirth generally obtained good scores. However, the dimensions related to the physical environment, the information received and decision making obtained lower scores. These aspects related to care could get higher scores when influenced by health care professionals. Therefore, measures for improvement should focus on them.

### 4.3. Proposed Measures for Improvement

In Spain, increasingly more studies are being conducted on patients’ satisfaction [36], but very few have been conducted on childbirth care since the Practical Guide was published by the Spanish Ministry of Health and Social Policy. Although validation studies have been done on questionnaires to evaluate satisfaction during childbirth, due to the methodology applied, proposals for improvements are scarce, and women’s perceived satisfaction has not been evaluated at different time points [37,38,39].

Regarding the physical environment, maternity wards must be designed to comply with specific standards as it is well-known that the environment influences the childbirth process [40]. There should be adequate lighting which can be adjusted; women should be provided with devices to facilitate movement; an individual bathroom in each room, preferably with large bathtubs where women can sit in hot water, as well as proper ventilation and enough privacy, should be offered.

From the received information, it is clear that healthcare professionals need to be more aware of what the childbirth experience means for women. Procedures that might be regarded by providers as routine and simple may be new and unfamiliar to women, which could generate feelings of fear. Accurate and easy information needs to be provided so that it is easily understood by every patient, regardless of their level of education. Professionals must confirm that every piece of information has been properly understood and show willingness to answer any question and solve any possible doubt the patient may have [41].

One of the main limitations found was the restricted amount of time to collect data. Not all of the potential care models that today exist in Spain are covered. Both centres that participated in this study are characterised by a substantial level of interventionism, so the impact of these outcomes on centres with less intervention still remains unknown.

## 5. Conclusions

Since the Clinical Practice Guide on Normal Childbirth Care [9] (currently under review) was published in Spain, studies that analyse the care received by women giving birth are needed. One important aspect is the satisfaction that women report about the care they receive, which was the main focal point of this work. This study is the first to be conducted on this matter, and it also evaluates satisfaction over one year in a forward-looking multicentre way in order to analyse the potential factors associated with quality. We conclude that users are generally satisfied with the care they receive in the hospitals that participated in this research. However, some aspects could be improved, such as the physical environment, the information that professionals provide throughout the process, and users’ participation in decision making.

In line with the results from previous studies developed in different environments [18], the fact that satisfaction scores may lower over time could be due to the normal evolution of the experience. Nevertheless, we cannot resign ourselves to this fact since there is a possibility (in line with the results from other studies on patients’ satisfaction [23,24]) that lowering scores are due to negative experiences during the puerperium that can even affect the results about the care received during childbirth. These negative experiences may include perineum pain or postpartum depression [22]. Under these circumstances, we highlight the need to conduct a follow-up of the care received by women during this period [42], so that the experience can be improved and the most usual problems that may arise can be overcome. In this way, it is possible that, by improving the postpartum experience, the satisfaction with the care received when giving birth is also maintained. Despite this, any work related to the assessment of the experience of giving birth must clearly indicate in which stage data are obtained in order to avoid not only over-evaluating, but also under-evaluating women’s opinions. It does not seem appropriate to strictly mark the time point when the satisfaction assessment should be done, as this could strengthen the belief that patients’ opinion is “real” in some cases, but “false” in others. Satisfaction evaluation from the point of view of the user is always subject to subjectivity, but the only required action is to assess this satisfaction degree by including a specific description of the time point at which the evaluation was done.

A future research line could imply satisfaction assessment at a higher level by including women from the maximum number of birth facilities/centres possible. This would show perceived quality for different alternatives (e.g., hospital births, birthing homes, home births).

We must focus on involving women in their own care. If women feel safe, supported and sufficiently informed, they will have the chance to play an active role in their childbirth process by making decisions. It is important to stress the importance that health education provided by health centres has in this regard. Primary Healthcare is an indisputable source of information, which must be a priority objective in working agendas. This way, users will have clearer expectations and a better idea of the options available to them, which will in turn help them make decisions in an easier way.

Regarding the possible complications during labour, women must be warned of the fact that, in these cases, it is essential to act swiftly and without hesitation, although the intervention is not what they would have decided at first. Professionals must establish a relationship with women founded on confidence and safety, in a way that women understand that, in case of complications, it is necessary to act according to the professionals’ indications so as to avoid negative perinatal outcomes. In this case, women will be less satisfied with the childbirth development, but they may still be satisfied with the care received.

In a childbirth without complications, and based on the information that professionals offer to women, patients could make decisions that better match their specific circumstances, without risking safety if they choose options considered less favourable for perinatal outcomes. Showing them that they have the last word in their childbirth process will help empower them and give them more say in one of the most significant and enriching experiences of their lives.

## Figures and Tables

**Table 1:** Socio-demographic characteristics (*n* and %), mean scores in the Intrapartal-Specific Quality from the Patient’s Perspective Questionnaire (QPP-I)scale, and statistical associations at each time point when the questionnaire was delivered.

Characteristic	*n* (%) (*n* = 248)	Mean Three Days (SD)	*p*	*n* (%) (*n* = 90)	Mean 15 days (SD)	*p*	*n* (%) (*n* = 83)	Mean one year (SD)	*p*
Age ranges (years) **			0.52			0.74		*	0.54
18–25	20 (8.1)	3.52 (0.42)		2 (2.2)	3.70 (0.22)		1 (1.2)	3.03 (-)	
26–35	169 (68.1)	3.43 (0.43)		68 (75.6)	3.38 (0.54)		59 (71.1)	3.26 (0.63)	
36–45	59 (23.8)	3.36 (0.52)		20 (22.2)	3.45 (0.42)		23 (27.7)	3.22 (0.48)	
Mean 32.35 (4.85)				32.20 (3.8)			33.02 (4.2)		
Place of birth *			***0.016***			0.67			0.40
Spain	215 (86.7)	3.39 (0.45)		79 (87.8)	3.41 (0.51)		75 (90.4)	3.23 (0.59)	
Foreigner	33 (13.3)	3.56 (0.44)		11 (12.2)	3.36 (0.52)		8 (9.6)	3.38 (0.62)	
Marital status *			0.15			0.67			0.99
Married	191 (77)	3.43 (0.46)		74 (82.2)	3.39 (0.51)		65 (78.3)	3.25 (0.60)	
Single	57 (23)	3.35 (0.42)		16 (17.8)	3.46 (0.48)		18 (21.7)	3.26 (0.57)	
Level of education *			***0.022***			0.25			***0.048***
Primary and Secondary	79 (31.9)	3.51 (0.40)		25 (27.8)	3.53 (0.35)		20 (24.1)	3.45 (0.60)	
Higher Education	169 (68.1)	3.37 (0.46)		65 (72.2)	3.35 (0.55)		63 (75.9)	3.18 (0.57)	
Parous *			0.65			0.97			0.58
Primiparous	122 (49.2)	3.43 (0.43)		44 (48.9)	3.39 (0.56)		40 (48.2)	3.20 (0.64)	
Multiparous	126 (50.8)	3.39 (0.48)		46 (51.1)	3.42 (0.45)		43 (51.8)	3.29 (0.54)	
Gestational age *			0.09			0.10			0.54
To term	239 (96.4)	3.41 (0.45)		84 (93.3)	3.42 (0.52)		80 (96.4)	3.25 (0.60)	
Preterm	9 (3.6)	3.13 (0.37)		6 (6.7)	3.20 (0.32)		3 (3.6)	3.15 (0.23)	
Type of feeding *			0.31			0.07			0.89
Breastfeeding	185 (74.6)	3.43 (0.44)		70 (77.8)	3.44 (0.50)		63 (75.9)	3.26 (0.57)	
Formula/Mixed	63 (25.4)	3.37 (0.46)		20 (22.2)	3.26 (0.52)		20 (24.1)	3.20 (0.64)	
Epidural *			0.27			0.35			***0.002***
Yes	208 (83.9)	3.39 (0.46)		79 (87.8)	3.43 (0.49)		73 (88)	3.27 (0.58)	
No	40 (16.1)	3.49 (0.42)		11 (12.2)	3.22 (0.61)		10 (12)	3.07 (0.67)	
Child birth mode *			***0.000***			***0.023***			***0.002***
Vaginal	212 (85.4)	3.47 (0.44)		74 (82.2)	3.45 (0.50)		68 (81.9)	3.34 (0.57)	
Emergency caesarean	36 (14.6)	3.09 (0.38)		16 (17.8)	3.19 (0.47)		15 (18.1)	2.84 (0.46)	
Initiation of labour *			***0.001***			0.21			0.26
Spontaneous	152 (61.3)	3.49 (0.41)		61 (67.8)	3.46 (0.45)		53 (63.9)	3.32 (0.55)	
Induction	96 (38.7)	3.30 (0.47)		29 (32.2)	3.29 (0.59)		30 (36.1)	3.13 (0.64)	
Newborn weight **			0.21			0.98			0.28
≤2499	3 (1.2)	3.25 (0.29)		2 (2.2)	3.55 (0.28)		3 (3.6)	3.43 (0.05)	
2500–3999	228 (92)	3.42 (0.45)		79 (87.8)	3.40 (0.551)		73 (88)	3.25 (0.59)	
≥4000	17 (6.8)	3.22 (0.49)		9 (10)	3.41 (0.55)		7 (8.4)	3.20 (0.74)	0.91
Antenatal classes *			0.29			0.95			0.88
Yes	120 (48.4)	3.39 (0.44)		42 (46.7)	3.42 (0.48)		39 (47)	3.23 (0.60)	
No	128 (51.6)	3.43 (0.45)		48 (53.3)	3.39 (0.53)		44 (53)	3.26 (0.58)	
Perineal trauma*			***0.035***			0.06			***0.04***
Entire/First degree tear	111 (44.8)	3.36 (0.42)		42 (46.7)	3.33 (0.46)		41 (49.4)	3.14 (0.54)	
Second degree/Episiot	137 (55.2)	3.45 (0.46)		48 (53.3)	3.47 (0.54)		42 (50.6)	3.35 (0.61)	
Duration of birth (hours) *			0.29			0.39			0.18
≤12 h	221 (89.1)	3.42 (0.45)		80 (88.9)	3.42 (0.50)		79 (95.2)	3.27 (0.58)	
>12 h	27 (10.9)	3.35 (0.42)		10 (11.1)	3.27 (0.58)		4 (4.8)	2.86 (0.71)	
Mean 393.15 (237.33)				390.14 (252.55)			351.12 (218.83)		

Bold and italics—significant results. * Mann–Whitney U test. ** Kruskal–Wallis test.

**Table 2:** Mean scores for each dimension at each time point when the questionnaire was delivered (left), and *p* values of the comparisons between each time point when the questionnaire was delivered (right).

Dimensions	Mean 3 Days (SD)	Mean 15 Days (SD)	Mean 1 Year (SD)	3 Days/15 Days	3 Days/1 Year	15 Days/1 Year
*1. The work of midwives*	3.73 (0.48)	3.65 (0.63)	3.55 (0.72)	0.217	***0.001***	***0.043***
*2. Second stage/evaluation birth*	3.55 (0.67)	3.52 (0.64)	3.42 (0.76)	0.454	***0.026***	3.78
*3. The work of doctors*	3.59 (0.58)	3.48 (0.69)	3.31 (0.77)	***0.033***	***0.002***	***0.04***
*4. The work of nursing assistants*	3.56 (0.70)	3.49 (0.83)	3.17 (0.97)	0.531	***0.000***	***0.006***
*5. Intervention/decision making*	2.61 (0.81)	2.85 (0.80)	2.70 (0.87)	***0.002***	***0.016***	0.542
*6. Accompaniment*	3.53 (0.60)	3.49 80.64)	3.32(0.79)	0.450	***0.033***	***0.014***
*7. Information*	3.35 (0.75)	3.35 (0.72)	3.12 (0.87)	0.788	0.080	0.113
*8. Facilities*	3.24 (0.90)	3.23 (0.95)	3.23 (0.80)	0.671	0.803	0.668
*9. Close relatives*	3.77 (0.51)	3.73 (0.56)	3.51 (0.80)	0.203	***0.002***	0.269
*10. Layout of the room*	3.19 (0.99)	3.19 (0.89)	3.07 (1.04)	0.378	0.689	0.949
*11. Feeling of safety*	3.69 (0.56)	3.61 (0.64)	3.40 (0.70)	0.111	***0.001***	0.084
*Total*	3.41 (0.45)	3.40 (0.50)	3.25 (0.58)	0.609	***0.003***	***0.004***

Bold and italics—significant results. Wilcoxon test.

**Table 3:** Binary regression logistic model, predictors of childbirth experience three days after birth (*n* = 248).

Model	Initial					Final				
Three Days	B	OR	95% CI		*p*-Value	B	OR	95% CI		*p*-Value
			Lower	Higher				Lower	Higher	
C-section	1.108	3.029	1.222	7.506	***0.017***	1.143	3.136	1.596	6.165	***0.001***
Initiation	0.828	2.289	1.233	4.251	***0.009***	0.775	2.171	1.199	3.931	***0.010***
Studies	0.377	1.458	0.750	2.836	0.267					
Duration	−0.21	0.980	0.390	2.459	0.965					
Perineum	−0.70	0.933	0.432	2.014	0.859					
Nationality	−0.769	0.463	0.164	1.309	0.147					
Epidural	0.485	1.625	0.716	3.689	0.246					
A. classes	−0.52	0.949	0.518	1.741	0.866					

B is the estimated parameter. Bold and italics—significant results. Defined variables: C-section (no = 1/yes = 0), initiation (spontaneous = 1/induction =0), studies (low = 1/high = 0), duration (≤12 h = 1/>12 h = 0), perineum (entire and first degree tear = 1/second degree tear and episiotomy = 0), nationality (X = 1/foreigner = 0), epidural (yes = 1/no = 0), A. classes (yes = 1/no = 0). *R*^2^= Initial Model: 0.101 (Cox and Snell), 0.147 (Nagelkerke); Final Model: 0.082 (Cox and Snell), 0.119 (Nagelkerke). The Final Model is significant ≥ Goodness of fit ≤0.000 (Omnibus). Final Model χ^2^ = 21.170.

**Table 4:** Binary regression logistic model, predictors of childbirth experience at 15 days after birth (*n* = 90).

Model	Initial					Final				
15 Days	B	OR	95% CI		*p*-Value	B	OR	95% CI		*p*-Value
			Lower	Higher				Lower	Higher	
C-section	1.047	2.849	0.628	12.926	0.175	1.185	3.272	1.043	10.266	***0.042***
Epidural	1.230	3.423	0.800	14.640	0.097	1.324	3.759	1.000	14.149	***0.050***
Studies	0.181	1.198	0.423	3.396	0.734					
Studies	0.305	1.357	0.417	4.417	0.612					
Duration	−0.009	0.991	0.195	5.027	0.991					
Perineum	−0.241	0.786	0.237	2.601	0.693					
Nationality	−0.093	0.911	0.192	4.321	0.907					
A. classes	−0.048	0.953	0.347	2,616	0.926					

Bold and italics—significant results. Defined variables: C-section (no = 1/yes = 0), initiation (spontaneous = 1/induction = 0), studies (low = 1/high = 0), duration (≤12 h = 1/>12 h = 0), perineum (entire and first degree tear = 1/second degree tear and episiotomy = 0), nationality (X = 1/foreigner = 0), epidural (yes = 1/no = 0), A. Classes (yes = 1/no = 0). *R*^2^= Initial Model: 0.088 (Cox and Snell), 0.126 (Nagelkerke); Final Model: 0.082 (Cox and Snell), 0.118 (Nagelkerke). The Final Model is significant ≥ Goodness of fit ≤0.05 (Omnibus). Final Model χ^2^ = 7.728.

**Table 5:** Binary regression logistic model, predictors of childbirth experience at one year after birth (*n* = 83).

Model	Initial					Final				
One Year	B	OR	95% CI		*p*-Value	B	OR	95% CI		*p*-Value
			Lower	Higher				Lower	Higher	
C-section	1.749	5.748	1.263	26.159	***0.024***	1.618	5.042	1.448	17.558	***0.011***
Epidural	0.673	1.960	0.427	8.996	0.387					
Initiation	−0.114	0.893	0.327	2.438	0.824					
Studies	0.421	1.524	0.462	5.024	0.489					
Duration	0.941	2.563	0.222	29.583	0.451					
Perineum	0.116	1.123	0.367	3.437	0.839					
Nationality	−0.960	0.383	0.063	2.341	0.299					
A. classes	−0.135	0.874	0.328	2.329	0.788					

Bold and italics—significant results. Defined variables: C-section (no = 1/yes = 0), initiation (spontaneous = 1/induction = 0), studies (low = 1/high = 0), duration (≤12 h = 1/>12 h = 0), perineum (entire and first degree tear = 1/second degree tear and episiotomy = 0), nationality (X = 1/foreigner = 0), epidural (yes = 1/no = 0), A. Classes (yes = 1/no = 0). *R*^2^ = Initial Model: 0.129 (Cox and Snell), 0.174 (Nagelkerke); Final Model: 0.084 (Cox and Snell), 0.114 (Nagelkerke). The Final Model is significant ≥ Goodness of fit ≤0.005 (Omnibus). Final Model χ^2^ = 7.323.
